# Mindfulness and negative emotions among Chinese college students: chain mediation effect of rumination and resilience

**DOI:** 10.3389/fpsyg.2023.1280663

**Published:** 2023-12-21

**Authors:** Keke Shi, Guoyan Feng, Qiyong Huang, Meilin Ye, Hongbo Cui

**Affiliations:** ^1^Guangzhou Xinhua University, Guangzhou, China; ^2^Zhangpeng Primary School, Dongguan, China; ^3^School of Education, Guangzhou University, Guangzhou, China; ^4^Mental Health Education and Counseling Center, Guangzhou University, Guangzhou, China

**Keywords:** mindfulness, rumination, resilience, negative emotions, college students

## Abstract

**Objective:**

This study examines the mediation effect of rumination and resilience between the relationship of mindfulness and negative emotions in Chinese college students.

**Method:**

A total of 3,038 college students (19.94 ± 1.10) were investigated by Mindfulness Attention Awareness Scale (MASS), Rumination Response Style Scale (RRS), Resilience Scale (RES) and Depression-anxiety-pressure scale (DASS-21), and the mediation analyses were conducted by adopting PROCESS macro in the SPSS software.

**Results:**

① Mindfulness was negatively associated with rumination and negative emotions (*r* = −0.69, −0.72; *P* < 0.01), and positively associated with resilience (*r* = 0.63, *P* < 0.01). Rumination was negatively associated with resilience (*r* = −0.59, *P* < 0.01), and positively associated with negative emotions (*r* = 0.83, *P* < 0.01). Resilience was negatively associated with negative emotions (*r* = −0.71, *P* < 0.01). ② Mindfulness can not only directly predict negative emotions (95%CI, −0.12~−0.09) but also affects negative emotions through three indirect paths: Rumination was a mediator (95%CI, −0.24~−0.20), resilience was a mediator (95%CI, −0.07~−0.06), and resilience and rumination were a chain mediator (95%CI, −0.04 ~ −0.03).

**Conclusion:**

Mindfulness not only influences negative emotions directly, bu*t* also through the mediating effect of rumination and resilience indirectly.

## 1 Introduction

As the pace of society accelerates and competition intensifies, people’s learning, work and life styles change accordingly. This leads to a more serious “involution phenomenon.” In addition, college students’ cognitive level and emotional and volitional development are not yet mature enough. This increases their psychological pressure and mental load, making it easy for them to experience negative emotions which can result in psychological and mental problems. Negative emotions occur in individuals who have stressful reactions, express pain or unpleasant perceptions (Chen et al., 2021), and are often manifested as a combination of depression, anxiety and stress (Ohayashi, 2012). Negative emotions are important indicators of mental health and can become the main risk factors for physical health. During the COVID-19 epidemic, 22.4% and 35.1% of college students reported symptoms of anxiety and depression, respectively (Kalkbrenner et al., 2021), with an overall detection rate of depression in adolescent populations climbing to 24.4%–32.4% (Gao et al., 2020). In China, the detection rate of depression among adolescents reached 24.6% in 2020 (Hankin, 2015), and in recent years, there has been a trend of prevalence of negative emotions such as depression, anxiety and stress (Ma, 2019). When individuals experience negative emotions and fail to resolve them in a timely manner, they are more susceptible to developing psychological problems and mental disorders, as well as an increased risk of self-injury or suicidal thoughts and behaviors (Lin et al., 2019). Given the substantial burden of negative emotions on individuals and society, it is crucial to understand the development and emotional problems of college students in order to promote preventive strategies and treatment.

Mindfulness is often defined as conscious and moment-to-moment awareness of present experience without judgment (Brown and Ryan, 2003; Baer, 2006). Through in-depth studies on mindfulness, many researchers have verified its effects on emotions. Previous studies have shown that mindfulness-based stress reduction training can improve negative emotions in people with depression. Mindfulness-based monitoring acceptance theory suggests that when individuals adopt an unacceptable attitude, their attention to physical sensations increases, which may strengthen negative emotions and thoughts associated with the experience. On the contrary, adopting an attitude of acceptance strengthens the relationship between attention and negative experience (Lindsay and Creswel, 2019). According to mindfulness theory, mindfulness enables individuals to focus on and understand their negative emotions in adversity, deal with them in a timely manner, find the meaning of events in life, actively respond to negative events (Garland et al., 2015), and mitigate harm caused by adversity. High levels of trait mindfulness can help individuals relieve anxiety, reduce the impact of stressful events on negative emotions (Zhou et al., 2017), lower the risk of depression (Zhang et al., 2022), and experience fewer subjective feelings of stress (Hertz et al., 2015) and negative emotions (Masumi and Alyson, 2017). Conversely, low levels of trait mindfulness do not contribute to an individual’s attention to and acceptance of negative emotions, which may lead to the long-term accumulation of negative emotions. Therefore, hypothesis 1 is proposed in this study: Mindfulness has a negative predictive effect on negative emotions.

Rumination is commonly described as an individual who remains influenced by negative life events, repeatedly thinking about the causes, consequences and feelings brought by these events (Nolen-Hoeksema, 1991). Reaction style theory suggests that human beings may react in different ways when they encounter various stressors related to life events. However, rumination is considered an inappropriate respond (Robinson and Alloy, 2003). A previous study has shown that mindfulness traits are not only significantly negatively correlated with rumination but also an important predictor of it (Wang et al., 2017). For instance, “non-responsiveness” on the Five Factor Scale of Positive Thinking were found to be negatively correlated with negative cognitive biases and rumination in healthy individuals after stress elicitation (Paul et al., 2013). Mindfulness can also promote an individual’s problem-solving abilities in adversity, reduce rumination, and help individuals adopt a more flexible and proactive approach to dealing with setbacks (Bajaj and Pande, 2016). According to the mindful emotion regulation model, mindfulness enhances cognitive control over negative immersive thinking by eliminating individuals’ automated negative appraisal process (Ramel et al., 2004). Meanwhile, the self-regulation execution function model suggests that rumination is a key factor in triggering negative emotions such as depression and anxiety in individuals (Nordahl et al., 2019). Domestic and international studies have found that rumination is significantly related to the severity of depression and cognitive function (Zhan and Yang, 2018), which is a cognitive susceptibility factor leading to depression (Nolen-Hoeksema et al., 2008). Individuals with high levels of rumination tend to compromise their problem-solving efficiency, have a longer duration of negative emotions, and a more serious tendency toward depression. Empirical studies have also shown that the higher levels of rumination are associated with higher levels of negative emotions such as depression and anxiety (McBride and Bagby, 2006; Grassia and Gibb, 2008). Conversely, the lower the level of rumination, the lower the level of negative emotions. Therefore, hypothesis 2 is proposed in this study: Rumination plays a mediating role between mindfulness and negative emotions.

Resilience, also known as psychological elasticity, refers to an individual’s ability to adapt flexibly to adversity and setbacks (Lazarus, 1993). Mindfulness has a significant predictive effect on mental resilience; increasing mindfulness levels can promote the development of students’ psychological resilience. Additionally, mindfulness training can effectively enhance cognitive function and the level of psychological resilience among practitioners (Senders et al., 2014; Zenner et al., 2014; Xu et al., 2016). A cognitive neuroscience study found that mindfulness may promote individuals’ psychological resilience by enhancing resting state functional connections between the dorsomedial prefrontal lobe and the anterior cingulate gyrus cortex during mediation (Kwak et al., 2019). This provides an intrinsic mechanism for how mindfulness affects rumination from a neuroscience perspective. Meanwhile, the risk-protective factor model posits that protective factors, such as resilience, can mitigate the impact of risk factors like rumination and prevent adverse psychological and behavioral outcomes, promoting healthy individual development. Studies have shown that encountering stressful events can stimulate resilience, which promotes effective coping with stress (Loh and Klug, 2012) and effectively reduces negative emotions such as depression and anxiety (Chen et al., 2019). Individuals with high resilience are able to respond to difficulties with a more positive attitude, reasonable beliefs, and flexible ways of maintaining lower levels of stress in adversity (Bajaj and Pande, 2016), thereby alleviating negative emotions associated with stress. Therefore, hypothesis 3 is proposed in this study: Resilience plays a mediating role between mindfulness and negative emotions.

Rumination and resilience can both play a mediating role between mindfulness and negative emotions, so what is the relationship between these two factors? Previous studies have found that there is a negative correlated between rumination and resilience (Chen et al., 2019). After experiencing stressful life events, individuals tend to engage in spontaneously repetitive thinking about those events, which can lead to continuous psychological and physiological arousal (Brosschot et al., 2006), depletion of cognitive resources, individual cognitive pattern disorders, and a propensity for negative thinking patterns that reduce psychological resilience. At the same time, individuals with reduced resilience may become less adaptable to negative events, which can make it difficult for them to recover from such events and increase the likelihood of experiencing negative emotions. Based on the above three hypotheses, this study concludes that individuals with low levels of mindfulness may tend to repeatedly ruminate on the causes, consequences and feelings brought about by stressful events. They may also struggle to eliminate cognitive biases, which reduces their internal psychological characteristics (such as resilience) and makes it difficult for them to adapt to adversity. This contributes to negative emotions such as depression and anxiety. Therefore, hypothesis 4 is proposed in this study: Mindfulness can affect negative emotions through the chain mediation of rumination and resilience.

Based on the literature above, more attention should be paid to the relationship between mindfulness and negative emotions. This study constructed a chain mediation model to examine the mediating role of rumination and resilience in the relationship between mindfulness and negative emotions among Chinese college students. Moreover, we proposed a model to examine the relationships among mindfulness, rumination, resilience and negative emotions.

## 2 Materials and methods

### 2.1 Participants

The convenience sampling method was employed to select students from colleges in Guangdong province of China to complete the questionnaires online from 1 May to 30 November 2022. In total, 3,038 valid questionnaires were obtained with an effective rate of 96.05%, while 125 unqualified ones were excluded. Among them, the mean age was 19.94 years (SD = 1.11, range = 17 ~ 25 years). The participants included 1,440 males (47.40%) and 1,598 females (52.60%). They completed a survey that included demographic variables, the Mindful Attention Awareness Scale (MASS), the Rumination Response Scale (RRS), the Resilience Scale (RES) and the Depression, Anxiety, and Stress Scale with 21 items (DASS-21). The purpose of this study was explained, and all respondents provided informed consent electronically before starting without any payment. Their privacy and anonymity were guaranteed.

### 2.2 Measurement variables

The data collected included socio-demographic characteristics of college students in Guangdong province, such as age and gender, as well as the information gathered from the four scales below.

#### 2.2.1 Mindful attention awareness scale

This scale was developed by Brown and Ryan (2003) and revised by Chen et al. (2012). It consists of 15 items rated on a six-point Likert scale, ranging from 1 (Always) to 6 (Never). A higher score indicated a greater level of mindfulness attention awareness. The Chinese version of the MAAS scale was used in this study, and it showed good internal consistency with a Cronbach’s α value of 0.94 in the present sample, confirming the good psychometric properties of the scale.

#### 2.2.2 Rumination response scale

This scale was compiled by Nolen-Hoeksema et al. (2008)and revised by Han and Yang (2009). It consists of 22 items rated on a four-point Likert scale ranging from 1 (Never) to 4 (Always), including three dimensions: symptom rumination, obsessive thinking, and reflective deep thinking. The higher the score, the more severe the rumination thinking is. The present study confirmed the good psychometric properties of the scale with a Cronbach’s of 0.94, indicating a good internal consistency.

#### 2.2.3 Resilience scale

This scale adopted the “Adolescent Resilience Scale” developed by Hu and Gan (2008), which consisted of two dimensions (personal strength dimension, including goal focus, emotional control and positive cognition; support dimension, including family support and interpersonal assistance). It consisted of 27 items rated on a five-point Likert scale, with scores ranging from 1 point (Complete consistently) to 5 (Complete inconsistent). A higher score meant that a higher level of resilience. For the present study, the Adolescent Resilience Scale was used, showing a good internal consistency (α = 0.93).

#### 2.2.4 Depression-anxiety-stress scale

This scale was compiled by Lovibond and Lovibond (1995) and later revised by Gong et al. (2010), with a total of 21 items, involving three types of factors of depression, anxiety and stress. All items were rated on four-point Likert scale ranging from 0 (nothing) to 3 (Most of the time). The higher the score, the more serious the corresponding negative emotional states such as depression, anxiety and stress are. The questionnaire has been proven to have good reliability and validity. In this study, the Cronbach’s α was 0.95.

### 2.3 Statistical analysis

The operation of data input was carried out by two people, and illogical or incorrect data were simultaneously checked. SPSS 25.0 and Hayes macro PROCESS in SPSS software were used for all data analyses. First, we conducted descriptive statistics of the socio-demographic characteristics among college students. Second, we conducted correlational analyses (using Spearman’s rank correlation coefficients) to examine whether mindfulness was related to other outcome variables (rumination, resilience and negative emotions) in the expected directions. Finally, multiple mediation analyses were conducted to test the mediating role of rumination and resilience in the relationship between mindfulness and negative emotions. We used 1,000 bootstrap samples, and bias were corrected at a 95% confidence interval (CI) to calculate the indirect effect of each variable. This indicated that the indirect effect were significant at *p* = 0.05 if the 95% confidence interval did not include 0 (Shrout and Bolger, 2002).

### 2.4 Common method biases

To eliminate the common method bias caused by the questionnaire survey, we conducted the Harman single-factor test to perform. The results of unrotated factor analysis showed that there were a total of 8 factors with characteristic roots were greater than 1. The first common factor explained only 35.98% (less than 40%) of the total variation, indicating that there were no serious common method deviation in this study.

## 3 Results

### 3.1 Descriptive statistics

Table 1 shows that demographic characteristics of the 3,038 Chinese college students who participated in this study. The mean age (SD) of the participants was 19.94 (1.10) years old. More than half (52.60%) of the participants were female. The median score (P25, P75) of the mindfulness, rumination, resilience and negative emotions of all participants was 4.00 (1.00, 6.00), 2.00 (1.00, 4.00), 3.44 (1.00, 5.00), and 1.67 (0.00, 3.00), respectively.

**Table 1:** Demographic characteristics of study participants.

Characteristic	Mean	Median (P_25_, P_75_)	% or SD
Age (years)	19.94		1.10
**Gender**			
Male	1,440		47.40
Female	1,598		52.60
Mindfulness (MASS score)	4.06	4.00 (1.00, 6.00)	1.11
Rumination (RRS score)	2.03	2.00 (1.00, 4.00)	0.60
Resilience (RES score)	3.45	3.44 (1.00, 5.00)	0.71
Negative emotions (DASS-21 score)	1.83	1.67 (0.00, 3.00)	0.65

MASS, Mindfulness Attention Awareness Scale; RRS, Rumination Response Scale; RES, Resilience Scale; DASS-21, Depression-anxiety-stress scale.

### 3.2 Correlations statistics of major study variables

The correlational analyses results of the four variables of mindfulness, rumination, resilience and negative emotions are shown in Table 2. Mindfulness was found to have a significant negative correlation with rumination and negative emotions (*r* = −0.69, −0.72; *p* < 0.01), while it had a significant positive correlation with resilience (*r* = 0.63, *p* < 0.01); Rumination was significantly negatively correlated with resilience (*r* = −0.59, *P* < 0.01), and it was significantly positively correlated with negative emotions (*r* = 0.83, *P* < 0.01). Additionally, resilience was significantly negatively correlated with negative emotions as well (*r* = −0.71, *P* < 0.01).

**Table 2:** Correlations analysis between the variables.

	1	2	3	4
1 Mindfulness	1			
2 Rumination	−0.69^**^	1		
3 Resilience	0.63^**^	−0.59^**^	1	
4 Negative emotions	−0.72^**^	0.83^**^	−0.71^**^	1

^*^p < 0.05, ^**^p < 0.01, ^***^p < 0.001.

### 3.3 The analysis of chain mediating effects of mindfulness and resilience

We conducted a mediation analysis using the SPSS PROCESS macro, version 3.4 (model 6), developed by Preacher and Hayes. Under the conditions of sampling 1,000 times, the mediating effect of rumination and resilience on mindfulness and negative emotions in college students was examined while controlling gender and age. Statistical significance level was set at *p* < 0.001 (two-tailed). Multiple regression analysis showed that mindfulness significantly negatively predicted rumination (*β* = −0.37, *p* < 0.001; Table 3). When both mindfulness and rumination were included as predictor variables, their effects on resilience were significant (*β* = 0.26, *P* < 0.001 and −0.35, *P* < 0.001). When mindfulness, rumination and resilience were included as predictor variables simultaneously, mindfulness and resilience negatively predicted negative emotions (*β* = −0.10, −0.24; *p* < 0.001), while rumination positively predicted negative emotions (*β* = 0.59, *p* < 0.001; Table 4).

**Table 3:** Regression analysis between the variables.

Regression equation		Global fit index			Significance of regression coefficient	
Outcome variable	Predictor variable	*R*	*R* ^2^	*F*	*β*	*t*
Rumination	Gender	0.69	0.48	918.39^***^	−0.004	−0.23
	Age				−0.04	−5.92^***^
	Mindfulness				−0.37	−50.15^***^
Resilience	Gender	0.68	0.47	660.82^***^	0.20	10.48^***^
	Age				0.02	2.54^*^
	Mindfulness				0.26	22.06^***^
	Rumination				−0.35	−16.13^***^
Negative emotions	Gender	0.88	0.77	2062.57^***^	−0.04	−3.83^***^
	Age				−0.0001	−0.03
	Mindfulness				−0.10	−13.36^***^
	Rumination				0.59	44.21^***^
	Resilience				−0.24	−22.36^***^

Adjusted age and gender, ^*^p < 0.05, ^**^p < 0.01, ^***^p < 0.001.

**Table 4:** The mediating effect of rumination and resilience between mindfulness and negative emotions.

	Mediating path	Effect	Boot SE	BootLLCI	BootLLCI	Proportion
Direct effect		−0.10	0.008	−0.12	−0.09	24.50%
Mediating effect	Total indirect effect	−0.31	0.010	−0.33	−0.29	75.50%
	X → M1 → Y	−0.22	0.009	−0.24	−0.20	52.77%
	X → M2 → Y	−0.06	0.004	−0.07	−0.06	15.25%
	X → M1 → M2 → Y	−0.03	0.002	−0.04	−0.03	7.50%
Total effect		−0.41	0.008	−0.43	−0.40	

X → M1 → Y: the indirect effects of mindfulness on negative emotions through rumination; X → M2 → Y: the indirect effects of mindfulness on negative emotions through resilience; X → M1 → M2 → Y: the indirect effects of mindfulness on negative emotions through rumination then resilience.

**Figure 1:**
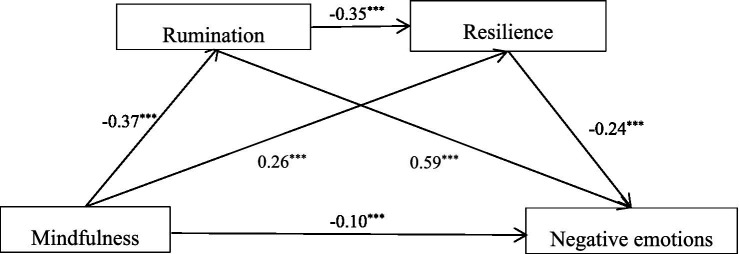
Model of mediating roles of rumination and resilience between mindfulness and negative emotions. ^***^p < 0.001.

As shown in Table 4, analysis of total indirect effect indicated that rumination and resilience partially mediate the relationship between mindfulness and negative emotions [Effect = −0.31, SE = 0.010, 95%CI (−0.33~−0.29)], accounting for 75.50% of the total effect. Meanwhile, when tested separately, the mediating paths were significant: the indirect effects of mindfulness on negative emotions through rumination (Effect = −0.22, SE = 0.009, 95%CI (−0.24~−0.20), excluding 0, and the mediating effect was significant), accounting for 52.77% of the total effect; the indirect effects of mindfulness on negative emotions through resilience (Effect = −0.06, SE = 0.004, 95%CI (−0.07~−0.06), excluding 0, and the mediating effect was significant), accounting for 15.25% of the total effect; the indirect effects of mindfulness on negative emotions through rumination then resilience (Effect = −0.03, SE = 0.002, 95%CI (−0.04~−0.03), excluding 0, and the mediating effect was significant), accounting for 7.5% of the total effect. The specific paths are presented in Figure 1. All the indices concerning the model effects are shown in Table 4.

## 4 Discussion

To the best of our knowledge, this is the first large-scale study to investigate the relationship between mindfulness, rumination, resilience and negative emotions among Chinese college students. At the same time, the study constructed a chain mediation model to comprehensively explores effects of cognitive factors (rumination), personality traits (mindfulness) and protective factors (resilience) on negative emotions. It mainly revealed the mechanism of rumination and resilience between mindfulness and negative emotions. The main aim of this study was to establish whether there was a relationship between mindfulness and negative emotions in Chinese college students, and whether resilience and rumination were a chain mediator of this relationship. The research of integration model is helpful in revealing the generation and development of negative emotions in college students. The research results can help us better understand the complex mechanism of mindfulness on negative emotions, which has certain theoretical and practical significance for strengthening the mental health education for Chinese college students.

### 4.1 The relationship of mindfulness and negative emotions

The research data showed that there was a significantly negative correlation between mindfulness and negative emotions, which validated hypothesis 1. Experience tells us that once stressful life events occur, established facts cannot be changed, and emotional reactions such as pain, anxiety, and depression do not help but make things worse. Mindfulness, as an important mechanism and method for regulating emotions, can prompt individuals to adopt an attitude of “allowing everything as it is” toward the present moment and adapt to the environment without judgment. Individuals with high levels of mindfulness can response to stressful events in a positive way. They may be aware of their current cognition, thinking and emotions in the present moment, accept current facts, and thus reduce problems such as distraction and inattention (Verhaeghen, 2021). They may also enhance their self-control and subsequently reduce the possibility of self-regulation failure (Leyland et al., 2019), resulting in e a lower level of negative emotions in subjective experience. This result is consistent with views of mindfulness monitoring and acceptance theory (Lindsay, 2017). That is, mindfulness intervention improves individuals’ cognitive levels, alleviates negative emotions and stress, and improves mental health through two core components: monitoring and acceptance.

### 4.2 The analysis of the chain mediating effects of mindfulness and resilience

A previous study supported the construction of a chain mediating model to explain the relationship between mindfulness and negative emotions, which elaborated on the mediating mechanism of rumination and resilience.

First, research data showed that rumination played a mediating role between mindfulness and negative emotions, which validated the hypothesis 2. Mindfulness significantly negatively predicted rumination, which was consistent with the research of Bajaj and Pande (2016). Mindfulness can promote individuals’ awareness and acceptance of their emotions, helping them to reduce rumination and frustration in adversity. Individuals with high levels of mindfulness tend to allocate more cognitive resources to the present moment, resulting fewer cognitive resources being devoted subsequently to the process of rumination, thus contributing to a reduction in rumination (Montgomery et al., 2016). At the same time, as a negative cognitive pattern and coping style, rumination is prone to causing individuals to dwell on stressful life events repeatedly and passively, leading to an inability to adapt in the face of difficulties and resulting in negative emotions such as depression, anxiety and stress. In general, individuals with low levels of mindfulness tend to exacerbate their repetitive thinking when faced with stressful life events. This caused them to indulge in negative events and increased resources for rumination process, which in turn leaded to further ruminative thinking and negative emotions. This result can inspire college educators to reduce negative emotions by guiding college students in carrying out positive attributions, making reasonable cognitive adjustments, and promoting positive rumination (Xuan et al., 2019).

Second, the research data also showed that resilience played a mediating role between mindfulness and negative emotions, validating hypothesis 3. The finding indicated that mindfulness significantly predicted resilience in a positive manner, which was consistent with the studies of Freligh and Debb (2019) and Wang and Kong (2020). Individuals with high levels of mindfulness were better able to focus on and understand their negative emotions during stressful life events, dealing with them in a timely manner. This reduced the damage caused by adversity and improved psychological resilience. Conversely, individuals with low levels of mindfulness may experience long-term accumulation of negative emotions due to their lack of attention and acceptance toward these emotions. This can lead to a reduction in psychological resilience as they attempt to minimize the harm caused by adversity. Meanwhile, resilience can help individuals cope better with stress, reduce the depletion of self-control resources in stress situations, and provide sufficient resources to facilitate better adjustment of negative emotions, thereby reducing the likelihood of experiencing negative emotions. In general, individuals with high levels of mindfulness are able to solve problems in a positive way, enhancing psychological resilience. Meanwhile, those with high levels of resilience tend to respond positively and overcome crises successfully by holding strong beliefs in the face of stress or adversity, ultimately freeing themselves from low moods and unpleasant experiences. Therefore, college educators can design mindfulness courses or implement mindfulness training to improve college students’ resilience and strengthen their willpower in solving complex difficulties or stressful events. This can help reduce the risk of negative emotions among college students.

Finally, when exploring the mechanism between mindfulness and negative emotions, the study also identified an important pathway consisting of chain mediation from rumination to resilience. Research showed that rumination may negatively predict resilience, and the result was consistent with the research conclusions of Ting Li et al. Individuals with high levels of rumination were more likely to be trapped in a negative thinking pattern, repeatedly enhancing themselves in the causes, effects and consequences of negative events. This increased their negative expectations for the future, creating a negative cycle that decreased their psychological resilience. To sum up, when faced with stressful life events, individuals with lower level of mindfulness tended to adopt negative attribution and cognitive approaches to solve problems. This can lead to a pattern of repetitive negative thinking, which decreased their resilience and made it difficult for them to maintain an adaptive level. Under such circumstances, individuals may experience negative stress and problems will be constantly worsened, resulting in negative emotions.

### 4.3 Limitation and prospect

Research limitation: The use of a convenience sample limits the universality of the results. This study employs the questionnaire survey method to examine the relationship between mindfulness, rumination, resilience and negative emotions in college students, which is a cross-sectional study and cannot well infer the causal association between the variables, so the results of the study have certain limitations. Furthermore, the current study did not investigate other potential factors such as perceived organizational support and work values. Research prospects: In the future, longitudinal follow-up studies or experimental research methods will be carried out, such as using the stochastic intercept cross-hysteresis model (RI-CLPM) to further explore the influence and mechanism of mindfulness on negative emotions in college students, and investigate the longitudinal relationship between cognitive regulation strategies and negative emotions more deeply, so as to maintain healthy psychological quality of college students.

## 5 Conclusion

This study explored the impact mechanism of the effect of mindfulness on negative emotions among Chinese college students. The main aim of the study was to determine whether there was a relationship between mindfulness and negative emotions in Chinese college students, and whether rumination and resilience were a chain mediator of this relationship. The regression model was utilized to synchronously examine the individual and chain mediating roles of mindfulness and negative emotions. The results indicated that a negative relationship exists between mindfulness and negative emotions in college students. Besides, resilience and rumination significantly influence negative emotions and played a mediating role in Chinese college students’ mindfulness and negative emotions. These results indicated the importance of both negative emotions and mindfulness. Other studies also showed that negative emotions, including depression and anxiety adversely affect academic performance (Asher et al., 2021). This can lead to increased college dropout rates and a decline in mental health. The findings from the present study have significant clinical implications for college psychologists who work with college students, as it provides evidence that lower levels of mindfulness lead to more negative rumination, lower resilience, and higher levels of negative emotions. These findings could also be used to design interventions that effectively treat and prevent emotions in college students when facing stressful life events. At the same time, it also indicates the importance of cultivating mindfulness among college students in dealing with stress and difficulties. This is because high levels of mindfulness can reduce negative emotions such as depression, anxiety and stress.

## Data availability statement

The original contributions presented in the study are included in the article/supplementary material, further inquiries can be directed to the corresponding author.

## Ethics statement

The studies involving humans were approved by the Institutional Review Board at Guangzhou Xinhua University. The studies were conducted in accordance with the local legislation and institutional requirements. The participants provided their written informed consent to participate in this study.

## Author contributions

KS: Conceptualization, Data curation, Formal analysis, Investigation, Methodology, Writing – original draft, Writing – review & editing. GF: Conceptualization, Data curation, Investigation, Methodology, Resources, Supervision, Writing – review & editing. QH: Conceptualization, Data curation, Investigation, Methodology, Writing – original draft. MY: Formal analysis, Investigation, Writing – original draft, Writing – review & editing. HC: Data curation, Investigation, Validation, Visualization, Writing – review & editing.
